# Assessment of African swine fever impact in Bulgaria with special focus on the East Balkan Swine

**DOI:** 10.3389/fvets.2025.1694525

**Published:** 2025-11-18

**Authors:** Elena Lazzaro, Dessislava Dimitrova, Radostina Doneva, Lisa Guardone, Alessandro Cristalli, Stefania Crovato, Jacopo Goracci, Hannes Bergmann, Vasco Menconi, Giorgia Angeloni

**Affiliations:** 1 Istituto Zooprofilattico Sperimentale delle Venezie, Legnaro, Italy; 2 Veterinari Senza Frontiere Italia, Legnaro, Italy; 3 Institute of Biodiversity and Ecosystem Research, Bulgarian Academy of Sciences, Sofia, Bulgaria; 4 Association for Breeding and Preserving of the East Balkan Swine, Shumen, Bulgaria; 5 Department of Veterinary Sciences, University of Pisa, Pisa, Italy; 6 Slow Food Italia, Bra, Italy; 7 Institute of Epidemiology, Friedrich-Loeffler-Institut, Greifswald, Germany

**Keywords:** African swine fever, small-scale farmers, indigenous breeds, East Balkan Swine, biodiversity conservation, biocultural heritage, food sovereignty

## Abstract

African Swine Fever (ASF) represents a significant threat to global pig production, due to its high lethality rate and the ability of the African Swine Fever Virus (ASFV) to persist in wild boar populations and the environment. In areas where small-scale pig farming is an important economic activity and a diverse source of protein, the disease also significantly affects nutritional security, food sovereignty and self-sufficiency. This study, conducted in Bulgaria, investigated the impact of ASF on small-scale pig farmers and East Balkan Swine farmers. A mixed-methods approach was employed, combining semi-structured interviews (*n* = 30), structured questionnaires (*n* = 10), and discussions with relevant authorities (*n* = 7), including farmers, health authorities and local veterinarians. The results highlight the vulnerability of traditional pig farming methods, with a significant focus on the East Balkan Swine, the last native pig breed in Bulgaria, whose population has been heavily affected by the disease.

## 1 Introduction

African Swine Fever (ASF) is a highly virulent viral hemorrhagic disease caused by the African Swine Fever Virus (ASFV), a large double-stranded DNA virus belonging to the genus *Asfivirus* (1). The disease primarily affects domestic pigs and wild boars, which are the sole susceptible hosts in Europe (2). As a result, the virus’s endemic presence in wild boar populations is a significant concern, posing a substantial threat to both pig production and wildlife management efforts (2, 3). The persistence of ASFV in swine populations is sustained by its stability in the environment and within animal carcasses, determining the “wild boar-habitat cycle” of Northern and Eastern Europe (2). Despite the identification of ASFV about a century ago, the absence of an effective vaccine has led to the adoption of control measures such as early detection, quarantine, stringent biosecurity protocols, restrictions on animal movements, and stamping out policies (1, 4–6). In regions where pig farming is a vital source of protein and supplementary income, ASF, through its high lethality in domestic pigs and the indirect effects of control measures such as culling campaigns and movement restrictions, has significantly impacted local economies, trade and pig production (4). While several studies in Africa and Asia have assessed the effects of ASF on smallholders and rural communities (7–17), evidence from Europe remains limited (18–23). In Eastern European countries, ASF outbreaks have notably affected backyard farms, which represent a key component of traditional agricultural systems (24–26). These farms are characterized by low and inadequate biosecurity measures and play a critical role in the persistence and spread of ASFV (18, 21, 22, 26–28).

**Figure 1:**
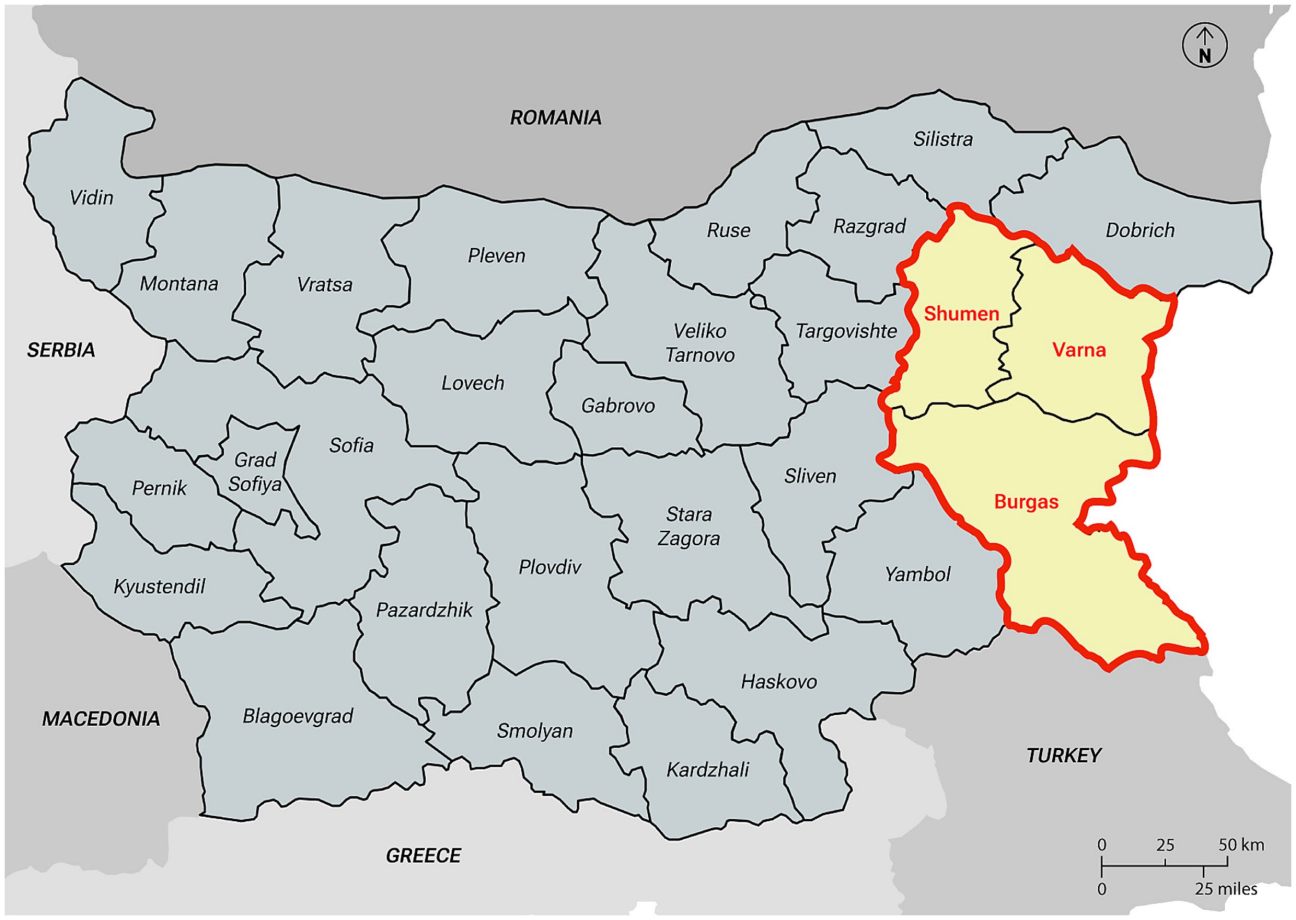
Map of the territories where EBS breed was officially farmed prior ASF (delimited in red).

**Figure 2:**
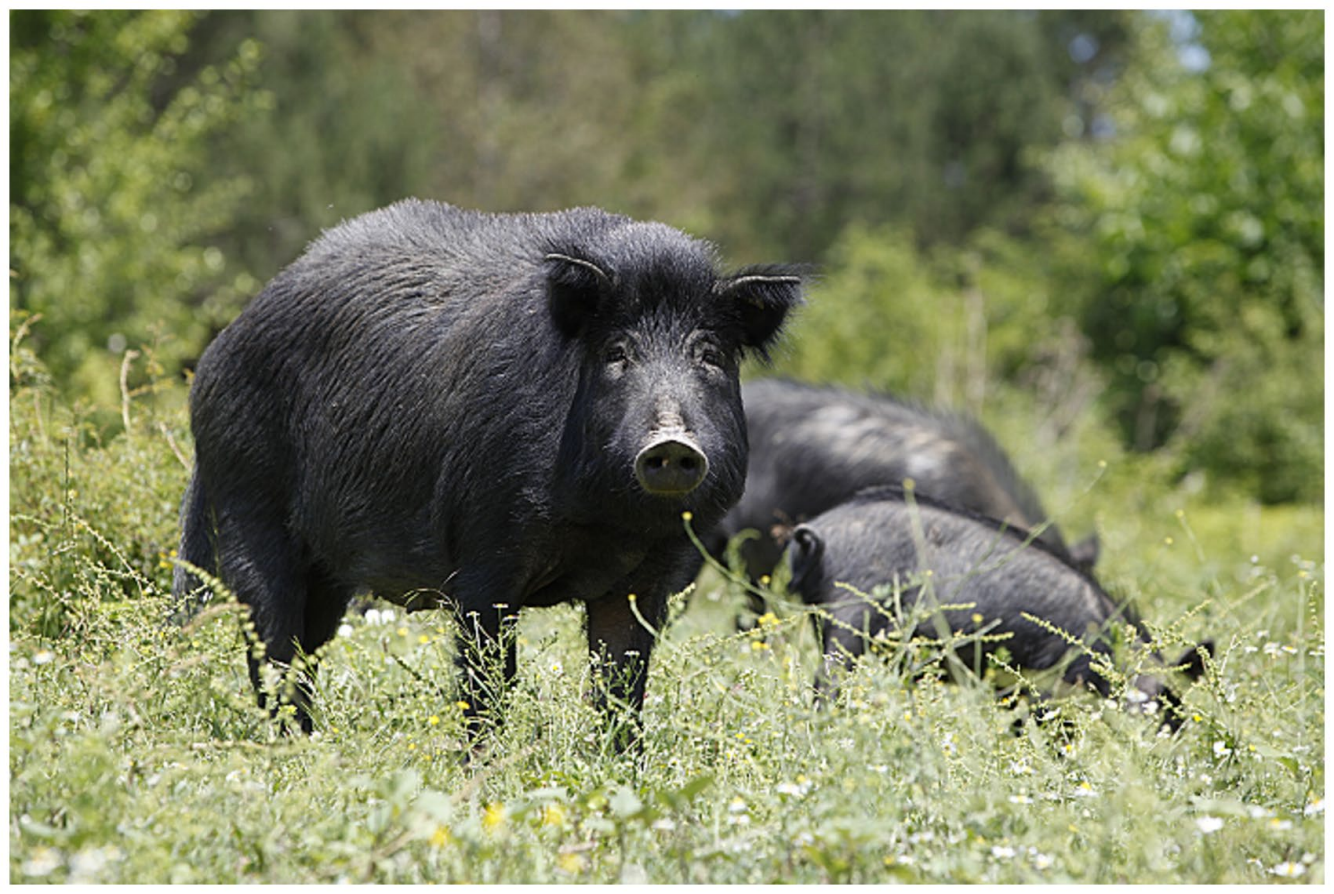
EBS pigs grazing in their traditional outdoor farming system. Image source: Kulev farm, 2017.

**Figure 3:**
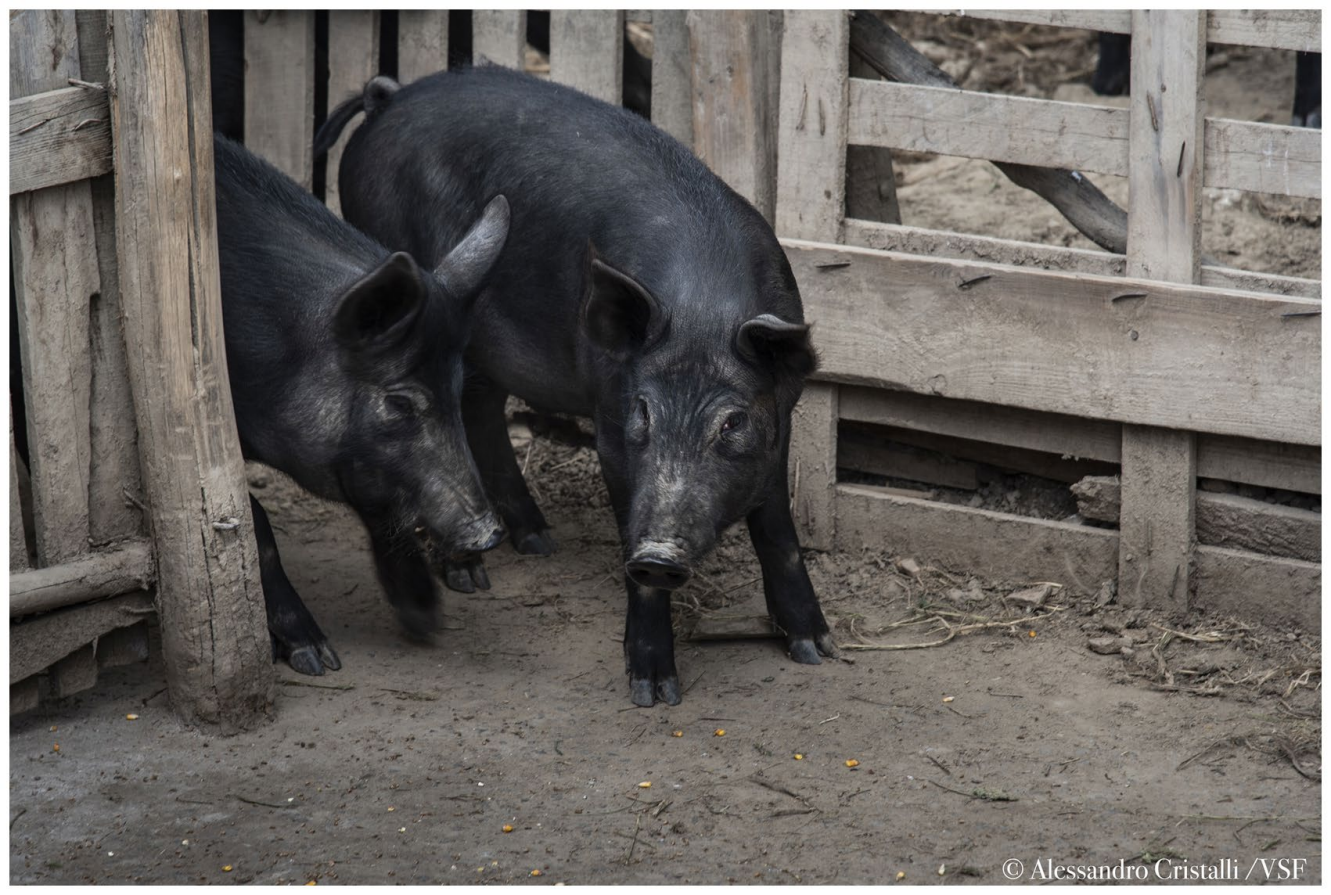
EBS pigs housed in fenced plots. Image source: Alessandro Cristalli, 2023.

Bulgaria, affected by ASF since August 2018 (25), exemplifies the challenges posed by insufficient control measures, which have facilitated the spread of ASFV across various regions. According to the Bulgarian Food Safety Agency (BFSA) in the European Food Safety Authority (EFSA) opinion on outdoor pig farming, ASF-related culling campaigns have led to the near-total destruction of the country’s backyard pig farming sector (29). Among Bulgaria’s most affected pig farming systems are the East Balkan Swine (EBS) farms, which add a significant chapter to the ASF narrative and serve as a case study relevant to many European contexts. The East Balkan Swine is designated as a Presidium by the Slow Food Foundation for Biodiversity, is the last Indigenous pig breed in Bulgaria and has a long tradition of outdoor farming in the forested areas of the eastern part of Bulgaria, including the regions of Burgas, Varna, and Shumen (Figure 1) (30). Since the arrival of ASF, EBS herds have suffered dramatic losses, placing the breed’s survival at serious risk. The epidemic has not only endangered this unique genetic resource but also disrupted traditional farming practices, suspended niche marketing of EBS products, and weakened the socio-economic fabric of the rural communities that depend on it. In fact, EBS farming supports local livelihoods and regional gastronomy, contributing to Bulgaria’s agrobiodiversity and traditional meat production. The breed’s high-quality meat, deeply connected to Bulgarian culinary traditions, has led to its official recognition by the Ministry of Agriculture and Food, and the establishment of a breeding association, the Association for Breeding and Preserving of the East Balkan Swine (ABPEBS), in 2006. Thanks to specific management practices and a tailored nutritional regime, EBS meat is notable for its high intramuscular fat content (5.38%), which imparts distinctive sensory attributes and rich flavor (30). Additionally, pigs raised in outdoor systems show a 4–6% increase in polyunsaturated fatty acids (PUFA) compared to those fed with conventional concentrate diets, further enhancing both the nutritional profile and the unique character of the meat (31–35). Before ASF, notably, EBS pigs were kept in large herds, feeding on pasture in the forests during the day (Figure 2), and housed indoors in simple wooden constructions during the night and for farrowing sows (30). This outdoor system not only suited the breed’s traditional husbandry requirements but also contributed to the high quality of their meat. Due to the complicated epizootic ASF situation in Bulgaria, free-range farming of EBS is currently prohibited as of 2019 for an indefinite time and the animals are required to be kept in enclosed premises or fenced plots for biosecurity reasons (Figure 3). The traditional forested regions, which would suit the unique husbandry requirements of the EBS breed and in which the keeping of EBS was officially permitted (Figure 1), are commonly occupied by other interest groups, such as hunters. Moreover, these traditional EBS husbandry grounds are often public property resources that require authorization from the competent authorities to permit fencing and thus meet key biosecurity requirements to keep EBS. These competing interests and fencing permit requirements hinder EBS breeders from accessing suitable grazing areas and then easily attaining the required biosafety level for disease protection of their pigs.

As ASF continues to spread in Europe, recognizing the cultural and economic importance of indigenous pig breeds such as EBS (*Cinta Senese*, Black Iberian, *Nero dei Nebrodi*) becomes essential for safeguarding genetic diversity and biocultural heritage, preventing the extinction of heritage lines and valuable genetic livestock resources (23, 36). This study focuses on analyzing the impact of ASF on small-scale breeders, with particular attention to the EBS breed in Bulgaria, highlighting the need for targeted actions that protect and support native pig breeds across Europe.

## 2 Materials and methods

### 2.1 Study area

**Figure 4:**
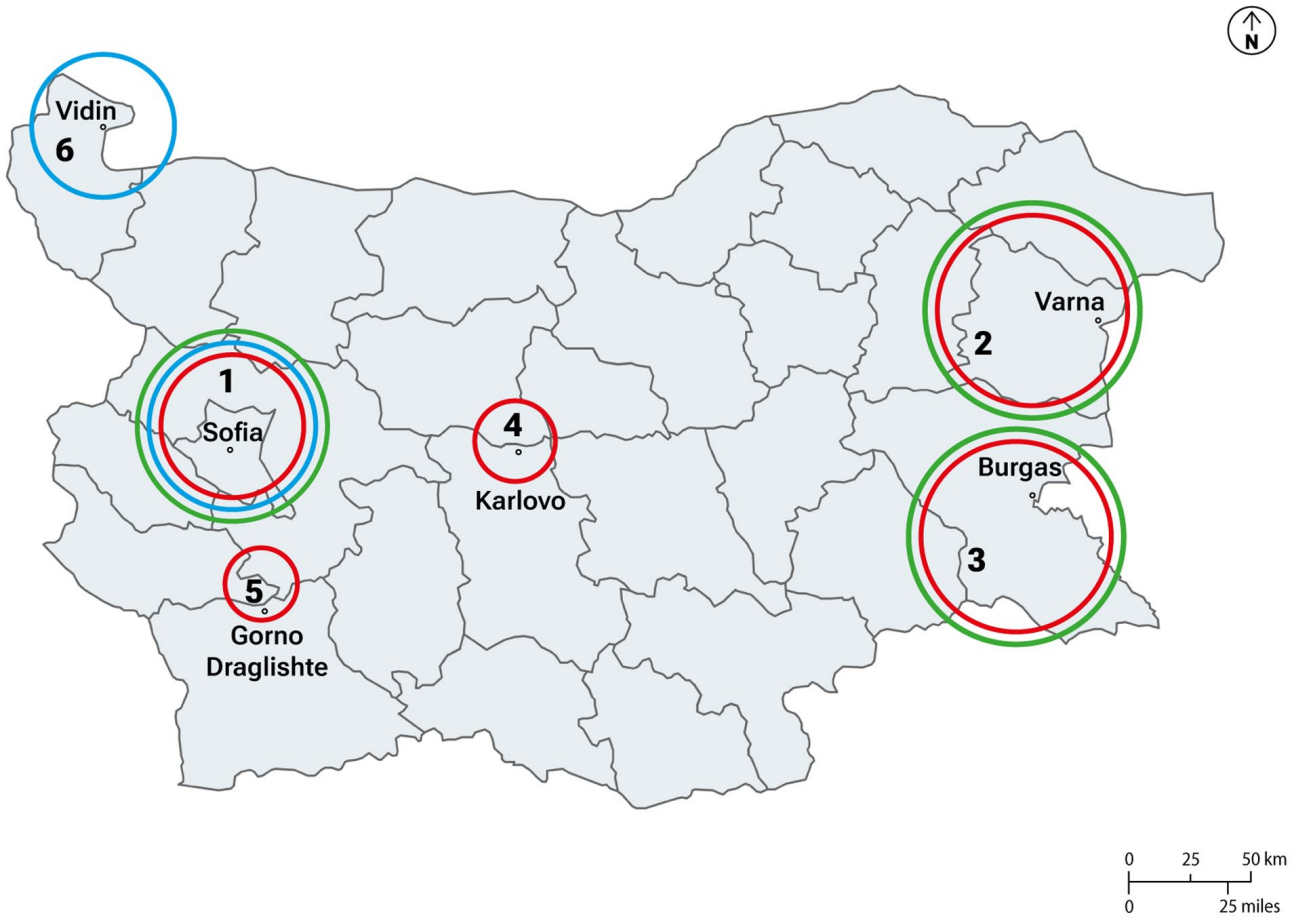
Map resume of the visits done during the first (in red) second (in blue) and third (in green) missions. (1) Sofia (2) Shumen; Varna (3) Burgas (4) Karlovo (5) Blagoevgrad; Gorno Draglishte (6) Vidin.

The study was conducted in Bulgaria (Figure 4), focusing on eastern regions where EBS pigs were officially farmed prior to the ASF outbreaks (Varna, Burgas and Shumen) (Figure 1). To provide a comprehensive overview, visits were also conducted in territories 1, 4, 5, and 6 (Sofia, Karlovo, Blagoevgrad, Gorno Draglishte and Vidin). ASF has led to a drastic decrease in the number of pig farms in Bulgaria. As of 2016, Bulgaria had approximately 29,930 backyard pig farms, a figure that fell to 6,859 by 2021, with the most significant decline occurring in 2019 - a 63.5% reduction compared to the previous year (37, 38). In fact, as of November 1, 2019, the number of pig farms had decreased by 73.8% compared to the previous year (37). This percentage highlights the severe impact ASF has had on the country’s pig farming sector, particularly smallholder and backyard farms, which are crucial to the livelihoods of many rural communities. Until 2018, EBS meat products were available on the market through the METRO retail chain and fine dining restaurants. At that time in 2018, BFSA declared 4,968 EBS pigs in 59 herds. The EBS case clearly demonstrates how ASF subsequently disrupted the advancement of marketing aimed at promoting traditional breeds, leading to the suspension of the procedure for entering “EBS meat” as a Protected Designation of Origin (PDO) product within the EU quality systems (personal communication: R. Doneva, president of ABPEBS). After ASF affected EBS herds, the Executive Agency for Selection and Reproduction in Animal Breeding (EASRAB) established six ex situ breeding groups, as part of two genealogical programs managed at artificial insemination stations in Kyustendil and Sliven, which are responsible for breeding and conserving genetic resources (39). The latest ministerial report recorded 1,056 EBS pigs culled from the end of 2019 to the end of 2023, leading to the breed’s classification as endangered (39). Currently, only 388 individuals remain, putting the population at risk of inbreeding and potential extinction (38, 39).

### 2.2 Study design and data collection

This study employed a mixed-methods approach, integrating multiple methodologies to gain a deeper understanding of the impact of ASF on Bulgarian farming, particularly among small-scale pig farmers (40). The preliminary work involved an extensive literature review, without historic time limits on the accessed records, concerning ASF and its implications in Bulgaria. This narrative review was based on sources, in English and Bulgarian languages, that included the PubMed literature database, available legislative documents and local newspaper archives. Search terms were selected around three main themes: African swine fever, smallholders, and East Balkan Swine. Semi-structured interviews were prepared based on the insights gathered from the literature data.

The research was conducted in three distinct phases (Table 1). The first phase involved semi-structured field interviews carried out in October 2022 (*n* = 14). The second phase comprised two meetings with relevant authorities (*n* = 7) to discuss policies and potential strategies, held in December 2022. The third phase, conducted between June and August 2023, involved additional semi-structured interviews (*n* = 16) and questionnaires (*n* = 10) that were developed based on the data and research collected during the previous phases (available in the Supplementary material).

**Table 1:** Stakeholders involved in semi-structured interviews and meetings, by number and category (2022–2023).

Year	Number of people	Category of stakeholders
2022	6	EBS and ex-EBS farmers
	4	Backyard and ex-backyard farmers
	1	President of the Association of Pig Breeders in Bulgaria (ABB)
	1	President of Bioselena Foundation
	1	President of Association for Breeding and Preserving of the East Balkan Swine (ABPEBS)
	1	President of the Executive Agency for Selection and Reproduction in Animal Breeding (EASRAB)
	3	Ministry of Agriculture and Food
	4	Risk Assessment Center on Food Chain
2023	7*	EBS and ex-EBS farmers
	4	Forestry professionals
	2*	Smyadovo’s Municipality
	3	Trakia University

*Participants who completed the structured questionnaire.

*These correspond to meetings with relevant authorities and are therefore excluded from the 30 semi-structured interviews.

As part of the EU Horizon 2020 DEFEND project, a workshop titled “Impact of ASF on backyards and minor breeds in Eastern EU Countries” was organized in Sofia on June 29–30, 2023, providing a platform for EBS and indigenous pig breed stakeholders to discuss strategies that could support traditional pig breeding systems threatened by the ASF epidemics in Europe (Table 2).

**Table 2:** Speakers and stakeholders contributing to the workshop in 2023.

Speakers	Country
Ministry of Agriculture and Food	Bulgaria
Risk Assessment Center on Food Chain	Bulgaria
Association for Breeding and Preserving of the East Balkan Swine (ABPEBS)	Bulgaria
Executive Agency for Selection and Reproduction in Animal Breeding (EASRAB)	Bulgaria
Slow Food Bulgaria	Bulgaria
Friedrich-Loeffler-Institut	Germany
Mangalitza pig breeders’ association	Hungary
Slow Food Italia	Italy
Veterinari Senza Frontiere Italia	Italy
Bazna pig breeders’ association	Romania
Scientific Veterinary Institute “Novi Sad”	Serbia
University of Castilla	Spain

The article reports data on a social research study based on the perception and opinion of breeders, no experimentation was carried out on humans or animals. The methodology of the EU Horizon 2020 DEFEND project No. 773701, which was used during this study involving participants in research interviews, was reviewed and approved by the Ethics Committee at the European University Institute (EUI). To comply with the General Data Protection Regulation (EU) 2016/679, a privacy agreement was requested from all participants involved in the research activities, and breeders signed a paper document before the interview began. In the introduction section of the questionnaires, the purpose of the survey was explained, and written informed consent to participate in this study was obtained from all participants.

### 2.3 Phase I and III—semi-structured interviews

A total of 30 semi-structured interviews were conducted (see Tables 1, 3). In 2022, 14 interviews involved EBS and ex-EBS farmers, backyard and ex-backyard farmers, and representatives of agricultural associations. In 2023, 16 interviews were conducted with EBS and ex-EBS farmers, forestry professionals, municipal representatives, and university professors. Interviews were conducted in the relevant areas for each group (Figure 4): EBS and ex-EBS farmers in EBS sites (sites 2 and 3), and non-EBS stakeholders in their respective locations (sites 1, 4, 5, 6). Each interview lasted between 1 and 2 h. The interviews focused on gaining insight into farmers’ perceptions of ASF, including its impact on their farms, practices, and daily routines. Additionally, the discussions aimed to understand the social and economic context in which these farmers operate, as well as their awareness and implementation of biosecurity measures. Questions were specifically designed to gather detailed information on pig-raising practices, the biosecurity measures that farmers deemed important and successfully implemented, and their views on ASF, including the main risks and potential factors (social, cultural, and ecological) contributing to its spread in their country. Example questions included: *“How has ASF affected your farm?,” “What measures do you take to prevent disease on your farm?”* and *“Which risks do you consider most important for the spread of ASF?.”* This qualitative approach, rooted in the methodologies described by Creswell and Poth (41), allowed for an in-depth exploration of the complex, context-specific factors shaping farmers’ experiences and practices.

**Table 3:** Overview of stakeholders, interview focus, and key themes identified in semi-structured interviews (*n* = 30).

Category of stakeholders	Number of interviewees	Interview focus	Main emerging themes
EBS and ex-EBS farmers	13	Impact and perception of ASF	Deep-rooted family and cultural tradition of EBS farming Economic and emotional impact of herd loss Financial barriers (fencing, lack of subsidies) Legal and logistical limitations to accessing public forest land Absence of slaughterhouses and formal meat market channels
Backyard and ex-backyard farmers	4	Impact and perception of ASF	Reluctance to resume farming activities following compulsory herd culling due to ASF
Associations (ABB; Bioselena Foundation; ABPEBS; EASRAB)	4	Policy advocacy and support measures for EBS breed conservation	Cultural and gastronomic value of EBS products Challenges posed by ASF and related restrictions Need for coordinated subsidies and support mechanisms Advocacy for official recognition, proper registration, and sustainable breeding programs Conservation strategies for EBS breed Compensation programs for backyard farmers
Trakia University	3	Genetic studies, breed conservation	Conducted studies on EBS genetics Provided scientific evidence for conservation programs Assisted in assessing population structure and inbreeding risks
Forestry professionals	4	Safeguarding traditional knowledge and local food heritage	State Enterprises manage six forest regions Fences permitted for breeding and implementing conservation plans for various game species in the territory, but EBS pigs remain excluded, requiring a separate concession from the Ministry of Agriculture
Smyadovo’s Municipality	2	Land access and public forest management for farm fencing	Local political willingness to preserve the breed and related traditions Opportunity to fence up municipal territories with a portion potentially dedicated to farming EBS pigs

The literature on qualitative research approach (41) highlights that field interviews and in-depth consultations with experts are valuable research tools for gaining a deeper understanding of the research focus and context, as well as respecting the appropriate ways of interacting with participants. In line with this approach, interviews were conducted in their natural setting, allowing for an in-depth exploration of the complex, context-specific factors that shape farmers’ experiences and practices.

### 2.4 Phase II—advocacy activities

During this phase, the focus expanded to include the local validation of previously collected data through in-depth consultations with Bulgarian veterinary authorities, including the Ministry of Agriculture and Food and the Risk Assessment Center on Food Chain.

Engaging in in-depth consultations with authorities was an important tool of qualitative approaches oriented to a participatory research activity. These have made it possible to gather detailed information at an expert level, as well as encourage discussion among stakeholders and increase knowledge about the issues addressed.

Emphasis was placed on developing practical, locally applicable biosecurity measures and solutions that could mitigate the impact of ASF on these farming communities. The dialogue also explored long-term solutions to strengthen the resilience of traditional farming practices, considering both the socio-economic and cultural significance of these systems in Bulgaria.

### 2.5 Phase III—questionnaires

The methodology included the administration of two structured questionnaires, supplemented by semi-structured interviews (42). Notably, seven participants who took part in the semi-structured interviews also completed the questionnaires (Table 1).

The questionnaires were structured based on the research and data collected during the 2022 missions. Two distinct versions of the questionnaire were developed:

(i) Farmers’ questionnaire

The questionnaire, comprising 36 questions primarily in closed-ended format, was divided into three sections: (1) demographic and holding data; (2) impact of ASF on the pig farms; (3) assessment of ASF perception.

(ii) Non-farmers’ questionnaire

The second questionnaire, consisting of seven questions, predominantly open-ended, was structured into two sections: (1) demographic and holding data; (2) assessment of ASF perception.

The questionnaires were pre-tested internally to assess the clarity of the questions and response options, the length of the questionnaire, and its technical implementation. Based on the feedback from the testers, improvements and corrections were made to refine the questionnaires. The final version of the questionnaires (available in the Supplementary material) was distributed both in physical form during the 2023 mission and online through a shared Google Form link in social network channels in July and August 2023. The online-distribution targeted individuals associated with the EBS case issue, such as those in related Facebook groups. Both questionnaires consisted of closed-ended questions (with single or multiple choices, and Yes/No questions) and open-ended questions, included to gather more detailed information. Some questions were mandatory, while others were optional. The questionnaires were estimated to take under 25 min to complete. The concluding section of both questionnaires included rating scale queries with five categories, intended to assess the perception of the effectiveness and applicability of various preventive measures utilized for preventing ASF.

### 2.6 Data analysis

All interviews were conducted in Bulgarian and transcribed in Microsoft Word in English. After the collection of the questionnaire, responses were translated into English and organized into a matrix using Microsoft Excel software. In this matrix, each row corresponded to an individual respondent, while each column represented a single question or item from the questionnaire, with responses coded accordingly (e.g., categorical values, binary coding for Yes/No, or numeric scales for rating questions). Once the dataset was structured, responses were cleaned, coded, and categorized before analysis. Using Excel functions such as filters and PivotTables, the data were systematically aggregated, frequencies and percentages were calculated, and PivotTables were used to explore associations between variables and to compare and facilitate the interpretation of results (please see Supplementary material). To protect the anonymity of the farmers, particularly given the small number of swine farmers involved, identifying details such as gender and age have been omitted from the quotes and removed from the Supplementary material.

## 3 Results

As delineated in the dedicated section, the findings of this study represent the outcome of a methodology that incorporates diverse research techniques. This comprehensive approach guarantees an exploration of the multifaceted aspects concerning ASF control and the conservation of traditional pig farming methods in Bulgaria.

### 3.1 Phase I and III—semi-structured interviews

The following sub-chapters present the data collected through the interviews (n = 30), focusing on insights into the impact of ASF and its perception in Bulgaria among various professionals. The participant categories and their numerical distribution are detailed in Table 1, which summarizes the types of respondents interviewed in 2022 and 2023, while Table 3 presents the interview focus and the main emerging themes. Special attention is given to the agricultural sector, specifically smallholders and EBS farmers. In this section, the most pertinent findings in relation to the study’s objectives are presented, emphasizing the key aspects that inform our understanding of ASF management and the challenges associated with sustaining traditional pig farming methods in the Bulgarian context.

#### 3.1.1 Challenges faced by EBS farmers: balancing tradition, genes conservation, economics and biosecurity

A solid and common trait found among the EBS farmers interviewed was the rooted tradition of EBS farming, which all of them have inherited from generation to generation, through which they have learned the breed’s peculiarities, contributing to the preservation of its uniqueness, the support of local livelihoods and food security, while also cultivating a lasting emotional bond with the animals. All but one of the interviewed farmers have lost their entire herds because of the direct and/or indirect consequences of the ASF epizootic, which caused an emotional impact on them. Beyond the emotional toll, EBS farmers faced significant economic repercussions. Prior to the ASF arrival, they relied on selling meat to retailers and restaurants. As stated by Radostina Doneva, president of ABPEBS, 60% of their annual income resulted from EBS meat sales. All the interviewed farmers were aware of the risks posed by ASF and had attended educational courses organized by the veterinary authorities at the municipal level, which focused on disease prevention measures such as disinfection and the use of electric fences. Despite their economic constraints, they made efforts to follow these recommendations. After ASF hit, EBS farmers emphasized the challenges of restarting their operations without a state-supported plan, as currently, national regulations do not provide for essential facilities, such as properly fenced areas. Yet such infrastructures are critical for reopening and registering farms, starting a legitimate EBS meat business, or becoming eligible for financial support.

In fact, adhering to the minimum biosecurity standards outlined in Regulation No. 6 (43), which sets the conditions and procedures for grazing herds of EBS and their crossbreeds, requires that EBS farmers have access to forestland that can be properly fenced. In this context, a consistent theme emerging from interviews was that for the establishment of an effective fencing system, economic support from the state is crucial, as was the case in other Member States (e.g., Executive decree n. 20882 of 2021 in Italy with a subsidy of 40% of expenses). Given that the recommended EBS minimum stocking density is four animals per hectare and the cost of installing a single row electric fence is 2,400 Lev (approximately 1,200 €) per hectare double-fenced, these expenses are financially challenging for farmers. Farmers point out the crucial necessity of subsidies to offset the initial restarting expenses, particularly given the protracted growth cycle of EBS pigs (typically reaching maturity by 2.5 years) and the heightened costs linked to stringent biosecurity protocols, including the obligatory utilization of commercial feed and the erection of protective fencing. In addition to financial considerations, there are legal constraints hindering EBS farmers from fencing forested areas, as these are publicly owned either by the State Enterprises, or by the Municipalities. Thus, farmers face limitations both economically and logistically. Farmers express deep concern not only about the loss of their animals, but also about the erosion of traditional knowledge and breeding expertise inherited from their ancestors, which are essential for the breed’s preservation and welfare.

##### 3.1.1.1 Smyadovo municipality case

The Smyadovo municipality in Shumen Province, Northeastern Bulgaria, represents a potential opportunity for the reestablishment of EBS farms in the area. The municipality mayor mentioned that the forest territories within its borders, although under the State Enterprise, are granted to the municipality, making it the automatic owner. She indicated the possibility of fencing these territories, totalling 2,000 hectares, with a portion potentially dedicated to farming EBS pigs. Currently, the farmers from the Smyadovo municipality host no more than 100 animals that can be farmed there. The mayor emphasized the significance of preserving the breed and the associated farming tradition deeply rooted in these territories.

#### 3.1.2 Backyard farming

The ex-backyard farmers have expressed that, while restarting their activities is theoretically possible, they have no intention of doing so due to a deep mistrust in the authorities and the fear of losing everything once again. Compensation for the culling of pigs, financed by the EU, provided 300 BGN (approximately 150 €) per culled pig, helping farmers partially offset their losses and cover the cost of disinfecting their facilities. In the Shumen region, the Association of Pig Breeders in Bulgaria (ABB) also contributed to compensating backyard farmers who were required to cull their animals near industrial farms to minimize the risk of infection before the first ASF outbreak. The president of ABB acknowledged that the permanent loss of backyard farms would lead to the disappearance of important food traditions and gastronomic knowledge, representing a significant cultural loss for the entire country.

#### 3.1.3 Wildlife and hunting framework

Bulgaria has approximately 160,000 officially registered hunters who are permitted to hunt wild boars in shared areas and sell wild boar meat (44). This activity is regulated by hunting laws, including the Law on Hunting and Preserving Game and the Regulations for Implementation of the Law on Hunting and Preserving Game.

The national Executive Forestry Agency, operating under the Ministry of Agriculture and Food, oversees six regional State Enterprises, managing territory, game breeding and control in line with hunting regulations. Despite the 2018 proposal by the North State Enterprise’s Director, EBS pigs are not included in the regulations related to forest territories, which now primarily address hunting. The Forest Agency has the authority to allocate specific areas for up to 15 years, such as those granted to hunters’ associations, to establish fenced areas for breeding and conserving various game species within the territory. For instance, approximately 200 hectares have been fenced in the North Eastern State Forest Enterprise for this purpose. However, since EBS pigs do not fall under forest territory regulations, their breeding requires general approval from the Ministry of Agriculture and Food.

### 3.2 Phase II—advocacy activities

A series of meetings with Bulgarian authorities was held to formulate strategies aimed at preventing the extinction of the EBS breed and, by extension, safeguarding the rich heritage of traditional farming practices. The key outcomes and insights from these discussions are summarized below:

1. Land use planning for EBS farming

Authorities emphasized the importance of identifying and designating areas within the breed’s natural habitat for EBS farming. These zones should comply with biosafety protocols and animal welfare standards, including providing adequate space for natural, breed-specific behaviors.

2. Financial support to farmers for proper fencing implementation

Although existing programs promote sustainable rural development and the conservation of EBS, it emerged that current frameworks do not consistently recognize fencing and other external biosecurity measures as eligible for financial support. It is important to highlight that competent authorities acknowledged the need to revise funding criteria to better support such measures.

3. Exemption from Regulation No. 10 (45)

For many years following the arrival of ASFV in Bulgaria, none of the slaughterhouses in the defined regions (Figure 1) accepted EBS for slaughter and meat processing, reflecting their perceived risk of cross-contamination from EBS to other pigs. This perception limited the availability of locally produced EBS meat and affected both the livelihoods of small-scale farmers and the food security of local communities. Additionally, Regulation No. 10, which provides specific guidelines for slaughterhouse use, stipulates that these facilities may only serve livestock affiliated with recognized producer groups or organizations. Unfortunately, EBS breeders are unable to obtain this status, as it requires proof of prior income from product sales—a condition that most EBS farmers, having lost their herds during ASF outbreaks, logically cannot meet. As a result, the proposal that emerged during the discussion was to allow EBS farmers to use slaughterhouses as producer groups, even if they have not generated verified revenue from previous sales. In parallel, the promotion of modular and mobile slaughterhouses was seen as a practical way to overcome accessibility challenges in remote areas. Another proposal focused on expanding the designated slaughter zones beyond the three defined in Regulation No. 6 (43).

4. Implementing a plan for conserving the genetic heritage of EBS *in situ* and *ex situ*

A comprehensive plan was discussed to ensure both in situ and ex-situ conservation of the EBS breed. This includes protecting existing populations, creating genetic repositories, and monitoring programs to preserve genetic diversity.

In April 2024, an amended version of Regulation No. 6 (43) came into effect, addressing the challenges faced by EBS farming. The revised regulation introduced specific amendments aimed at safeguarding the conservation of the breed, including the removal of restrictions on breeding within the three historically designated areas (Figure 1), allowing farming to expand into other regions of the country. Additionally, slaughtering was no longer confined to these areas, potentially increasing the number of accessible slaughterhouses.

### 3.3 Phase III—questionnaires

Ten respondents answered the questionnaires, collected both during the field mission (*n* = 7) and online (*n* = 3) (see Supplementary material). The results are presented according to the structure of the questionnaires. Please note that as only one questionnaire belongs to the (ii) non-farmer type, the described results pertain to the questionnaire intended for farmers (*n* = 9).

#### 3.3.1 Demographic and holding data

Most of the respondents were male, with the majority (87.5%) located in regions designated by Regulation No. 6. Over half of them owned EBS pig farms, though only one farm currently has registered EBS pigs. Among the remaining respondents, one operated a family farm (11–200 pigs), while two managed industrial farms (>1,000 pigs). Four of the six EBS farmers surveyed, including the one with the registered EBS farm, mentioned that they primarily raised EBS pigs as a supplementary source of income, providing quick cash when needed. The remaining two farmers considered EBS pig breeding their primary source of income. Before the onset of ASF, all EBS farmers used the traditional free-range system for pig breeding. The registered farmer successfully installed wooden fences, even without a concrete foundation. Except for two industrial farmer who used commercial feed, the others reported feeding their animals with fodder, grass, grain, and broken rice. Additionally, the diet of EBS pigs primarily relied on forest products like acorns, grass, shrubs, roots, worms, truffles, mushrooms, and wild berries, especially before the ASF epidemics, when the pigs had access to the forest.

#### 3.3.2 Impact of ASF on the EBS pig farms

Most of the surveyed farmers identified restrictions on animal movement as the most impactful control measure against ASF. When considering the direct effects of ASF, limitations on trade and economic losses were seen as the most significant adverse consequences for their farms, with 88.9% (*n* = 8) and 55.6% (*n* = 5) of respondents citing these issues, respectively. The majority of respondents (*n* = 8) had undergone specialized training courses conducted by veterinary authorities, aimed at preventing ASF on their farms. These courses, complemented by awareness campaign materials like brochures, posters, and coverage in local newspapers and television news, represented the main sources of information.

Notably, 87.5% (*n* = 7) of the respondents did not receive subsidies for implementing preventive measures. Despite this, all the respondents undertook specific preventive actions, including disinfection and, to a lesser extent, the installation of fences. Despite their proactive efforts to prevent infection, ASF directly affected the (*n* = 5) of the total respondents, with 66.7% (*n* = 4) of EBS farmers reporting infection on their farms. Of those affected, only 60% (*n* = 3) received subsidies, which were often insufficient to cover the losses they incurred.

#### 3.3.3 Assessment of ASF perception

Regarding the dynamics of ASF spread, 55.6% (*n* = 5) of respondents identified direct contact with infected animals as the most likely way for the disease to enter a farm, while a smaller group (22.2%, *n* = 2) considered insects as potential vectors. Respondents primarily attributed the spread of ASF within the country to wild boars and human movement. Based on the perception rating scale used to assess preventive measures, respondents considered hygienic-sanitary actions, such as washing and changing clothes and footwear, banning the use of food waste and outdoor feeding, and maintaining cleanliness and disinfection on the farm as both the most effective and feasible actions (mean > 3.5). In contrast, although structural and managerial measures (e.g., fencing, farm quarantine and restricting movements of people and vehicles into the farm) were also recognized as effective, respondents found them more challenging to implement. Measures perceived as less effective and applicable (mean < 2.5) include using only commercial feed, rodent control and destroying feed and bedding materials on the farm.

## 4 Discussion

African swine fever has a devastating impact on the Bulgarian EBS farms, leading to near-total herd losses and threatening the conservation of this swine species (38, 39). Economically, farmers suffered significant income losses, with EBS meat previously accounting for up to 60% of annual earnings. Restarting farms is hindered by the high costs of biosecurity measures and fencing, as well as legal restrictions on using forested land. On the other hand, backyard farmers faced similar challenges and mistrust of authorities. Despite all interviewed EBS farmers attending ASF prevention courses and implementing biosecurity measures, 66.7% of them were unable to primarily prevent infection, highlighting the urgent need for targeted state support to sustain both the socio-economic and cultural viability of EBS farming in Bulgaria. This study highlights critical issues and proposes targeted actions to address current challenges. A major concern identified is the financial and legal obstacles to restarting EBS breeding, compounded by the lack of a national strategy and state funds for adapting to necessary prevention and control measures, such as fencing. In this context, both offering economic support and providing effective educational initiatives, while balancing the measures and evaluating their applicability, are crucial for fostering trust and preventing the adoption of illegal farming practices. In fact, the absence of tangible assistance has led to widespread disillusionment with authorities, risking an increase in inappropriate breeding practices and underreporting of the disease. Structured legal and economic support is particularly vital in alleviating financial burdens following crises like ASF outbreaks, enabling farmers to adopt necessary biosecurity measures without financial strain. Transparent and accessible support programs not only help farmers but also reinforce the credibility and reliability of authorities as partners in agricultural stewardship. By integrating education with tangible economic support and tailored control measures, authorities can empower farmers to comply with regulations, adopt sustainable practices, and contribute to the overall health and integrity of the farming sector. The situation with backyard farmers is especially dire, as in Bulgaria the loss of trust in state assistance has led to the abandonment of this type of farming. This loss of livelihood is also a significant blow to the preservation of cultural heritage, as backyard farming is deeply intertwined with traditional methods and societal identities, while also serving as a crucial source of meat and income in rural areas (25). The case of EBS in Bulgaria highlights the broader challenges facing indigenous pig breeds across Europe, which are not only ecologically significant but also culturally and socio-economically important (22, 23). These outdoor breeds, such as the Iberian pigs, play a critical role in conserving agroforestry ecosystems and producing prized pork products (22, 29). Outdoor and organic farming systems, which are central to the production of unique local pork products like Italian *Cinta Senese* and Corsican dry-cured meats, are also gaining popularity in Western Europe due to the increasing demand for environmentally and socially sustainable goods (23, 46). However, these systems often have lower levels of biosecurity, contributing to the spread of diseases like ASF (22). To address these challenges, discussions with Bulgarian authorities have emphasized the urgent need for strategies to prevent the extinction of the EBS breed and generally to preserve traditional farming practices. This includes the implementation of preventive actions, sustainability strategies, and appropriate subsidies, particularly during epidemic outbreaks. Collaborative efforts between stakeholders and policymakers are crucial, and comparisons with other European realities highlight the necessity to apply tailored policies and EU derogations for ASF control measures that emphasize the preservation of indigenous breeds. Preserving intangible cultural heritage, including traditional food practices and knowledge transfer, is vital for maintaining societal identities and supporting economic and social progress. Despite their ecological significance and contribution to biodiversity, indigenous pig breeds across Europe confront a significant decline, emblematic of broader challenges arising from industrialization, diminishing the economic role of rural areas and other socio-economic changes, exacerbated by epizootics like ASF (36). The small sample size of the survey, comprising only 10 respondents to the questionnaires, may have limited the generalizability of the results. Moreover, the reliance on self-reported data from farmers and authorities may have introduced some biases, as responses could have been influenced by personal perspectives or recall inaccuracies. However, this study reveals severe challenges faced by farmers, ranging from economic impacts to the emotional strain of losing entire herds and their usual livelihood. Critically, it highlights the lack of comprehensive indicators to measure the socio-economic and cultural value of these breeds, underscoring the need for their development to drive advocacy and enable meaningful change. Aligned with EU policies such as the Green Deal, prioritizing sustainability in meat production is critical not only for meeting contemporary environmental and ethical standards but also for ensuring resilience and adaptability in the face of ongoing climate change. Indigenous pig breeds, with their diverse genetic traits, play a pivotal role in this process.

In conclusion, this study highlights the wide range of challenges ASF poses in Bulgaria, focusing on the impact on smallholder pig farmers and the EBS. Our findings illustrate how the ASF occurrence has decimated traditional and EBS small-scale farms and threatened traditional farming systems. Despite the awareness of the disease and efforts to comply with biosecurity measures, the lack of state recovery plans and restrictive regulations have severely hampered Bulgarian farmers. Moreover, the destruction of Bulgaria’s backyard farming production represents the progressive erosion of traditional knowledge. The Bulgarian case highlights the urgency of specific policy frameworks that recognize and support traditional and small-scale farming systems. This should include flexible regulatory adaptations and economic incentives for adopting biosecurity infrastructures. Preserving endangered breeds such as EBS is a biodiversity matter and a socio-cultural challenge. Coordinated efforts at the European level are essential to ensure that ASF control measures align with the sustainability of traditional livestock systems and the countries depending on them, with the aim of safeguarding invaluable cultural and genetic resources for future generations.

## Data Availability

The raw data supporting the conclusions of this article will be made available by the authors, without undue reservation.
