# Supplementary material for: Assessment of African swine fever impact in Bulgaria with special focus on the East Balkan Swine
Source: Front Vet Sci. 2025 Nov 18;12:1694525. doi: 10.3389/fvets.2025.1694525 (PMC12671380; doi:10.3389/fvets.2025.1694525)
Supplement: Supplementary file 1 [file Table_1.DOCX]

Supplementary Material

# Farmers’ questionnaire template (English version)

**IMPACT OF ASF ON EBS FARMS AND BACKYARDS IN BULGARIA**

**INDIVIDUAL INTERVIEWS**

This questionnaire aims to collect local information on the effect of the current EU Regulations and policies related to ASF on traditional and smallholder farmers. I’m Elena Lazzaro, and I work as a veterinarian in a Public Institute of Research in Italy (Istituto Zooprofilattico Sperimentale delle Venezie). I will collect this data thanks to a project financed by the Italian Society of Swine Farming and Pathology, in collaboration with the Institute of Biodiversity and Ecosystem Research.

Data collected will be used according to the Regulation (EU) N ° 2018/1725

By agreeing with The EU Regulation privacy statement and answering this questionnaire, you agree that your answers are used anonymously in the technical report and related peer-review publication that will be produced.

- Yes, I Agree
- No, I Don't Agree

**Demographic and Holding Data**

***Data of participants and farm general characteristics***

Email ____________________________

Age _______________

Sex _______________

Village _______________

1. **Present number of pigs in your farm**

- ≤ 3
- 3-10
- 10-200
- 201-1000
- ≥1000
- None

1. **Which is/was your farm type?**

- East Balkan Swine farm
  - EBS pigs registered in the herdbook
  - Crossbreading of EBS
  - Both. Indicate the proportion ___________________________________________
- Personal holding (backyard) (=*up to three fattening pigs other than sows and uncastrated boars*)
- Family farm (=*up to 10 sows and their offspring, but not more than 200 pigs in total*)
- Industrial farm

**2a. How many people are part of your household?**

| **Category** | **Number** |
| --- | --- |
| **Kids <5 years old** |  |
| **Kids <18 years old** |  |
| **Adults <45 years old** |  |
| **Adults >45 years old** |  |
| **Adults >65 years old** |  |

1. **For which purposes do you raise/have raised pigs**

- For self-consumption
- Occasional additional income (quick cash when needed)
- Main income generation activity /market oriented (sale)

1. **What type of housing system did/do you have?**

- Free range system unfenced
- Free range system fenced
  - How did build the fences? ______________________________
  - Which type of fences?
    - Wood fences/without concrete base
    - Wood fences/cemented floor
- Full time housed system
- Elevated wooden floor
- Concrete building
- Other ________________________________

1. **How do you feed pigs?**

- Swill (food scraps, kitchen leftovers)
  - Do you cook or boil it before feeding? Yes/Sometimes/Always
- Fodder, grass, grain, broken rice, etc.
- Forest products. Which ones? ________________________________
- Commercial feed
- Other ________________________________

1. **Other livestock. Do you have other animals on the same farm?** **If yes, please specify which.**

- Yes __________________________________________________
- No

1. **Do you or your family member practise forest activities (e.g. hunting, mushroom picking...)? If yes, specify for which aim and the weekly frequency.**
   - Yes __________________________________________________
   - No

**A – IMPACT OF ASF ON YOUR PIG FARM (just farmers hit by ASF)**

1. **Did you have cases of ASF in your farm? If yes, specify the month and the year.**

- Yes ____________________________________________________________________
- No

**1.1 If yes, did you receive subsidies to compensate your economic losses?**

**
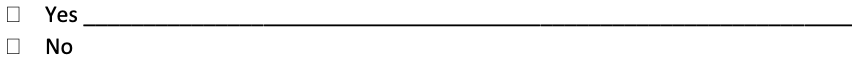
**

**1.1a If yes, were the subsidies sufficient to cover the losses? How much money have you received? Which share of the pig commercial value was reimbursed?**

__________________________________________________________________________________________________________________________________________

**1.1b If no, why did you not receive subsidies?**

__________________________________________________________________________________________________________________________________________

1. **Did you have to cull your animals because you were close to an ASF outbreak? If yes, specify the month and the year.**

- Yes ____________________________________________________________________
- No

1. **Have you adopted specific preventive measures in your farm related to ASF? If yes, please specify which (e.g. fences), the reason and when.**

- Yes ____________________________________________________________________
- No

**3.1 Did you receive subsidies to sustain the implementation of the preventive measures?**

**
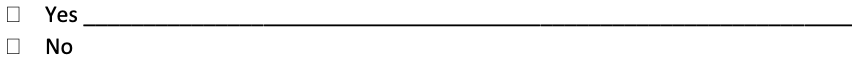
**

**3.1a If yes, were the subsidies sufficient? How much money have you received?**

______________________________________________________________

**3.1b If no, why did you not receive subsidies?**

______________________________________________________________

1. **Have you followed training courses to prevent ASF in your farm?**

- Yes
- No

1. **Which was your main source of information for ASF and to know how to act? (Multiple choices)**

- Vet authorities’ educational courses
- Brochures and posters of the awareness campaign
- Local newspapers
- Radio
- TV news
- Social networks
- Other (please specify) _____________________________________________________

1. **Which were the restrictions that, in your opinion, have the major impact on your farm? Please motivate your answer.**

______________________________________________________________________________________________________________________________________________________________________________________________________________________________________________________

1. **Which were the negative consequences of ASF impact on your farm? (Multiple choices)**

- Economic losses for the farm
- People lose job
- Unfair compensation by the government
- Restrictions on trade
- Other (please specify) ______________________________________________________

1. **Does ASF have an impact on the number of heads in your farm?**

- Yes
  - Can you quantify it? _______________________________________________

|  | **Adults** | | **Piglets** | |
| --- | --- | --- | --- | --- |
|  | **Male** | **Female** | **Male** | **Female** |
| How many pigs did you have before ASF arrived? |  |  |  |  |
| How many pigs do you have now? |  |  |  |  |

- No

1. **Did you change the type of animals or the pig breed farmed because of ASF? If yes, please specify which animals/breeds you introduced.**
   - Yes _____________________________________________________________________
   - No
2. **Did you change your habits related to the forest (e.g. hunting, mushrooms picking...) because of ASF consequences? If yes, specify in what way.**
   - Yes _____________________________________________________________________
   - No

**B – ASSESSMENT OF ASF PERCEPTION**

1. **Which are the clinical signs that make you suspect of ASF?**

____________________________________________________________________________________________________________________________________________________________________

1. **Which are the post-mortem signs that make you suspect of ASF?**

____________________________________________________________________________________________________________________________________________________________________

1. **In which cases would you call a veterinarian for your pigs?**

- One pig not eating well/looking sick
- Several pigs not eating well/looking sick
- Increased mortality
- Sow not having piglets
- Preventive treatment such as vaccination and/or deworming
- Other _________________________________________________________________________

1. **If you observe reduced heating, redness of skin and mortalities in your pig herd, what would you do?**

- Wait few days to see if the pigs improve
- Treat the pigs with antibiotics
- Sell the pigs as soon as possible
- Call the veterinarian services
- Other _________________________________________________________________________

1. **If you suspect an ASF outbreak in your farm, what would you do?**

- Wait few days before reporting it to avoid a false report
- Wait few days before reporting it to have time to sell the healthy pigs
- Report it as soon as possible even if might be a false case
- Other _________________________________________________________________________

1. **Which way of transmission do you consider the most effective for ASF introduction in a farm?**

____________________________________________________________________________________________________________________________________________________________________

1. **Which way of transmission do you consider the most responsible for ASF spread in your country?**

____________________________________________________________________________________________________________________________________________________________________

1. **Do you think that social factors (e.g. movement of people/animals, family sharing of food meat products, migration, travel migrants) could have played a role in ASF spread? Please explain your opinion.**

____________________________________________________________________________________________________________________________________________________________________

1. **How much do you consider effective and applicable these preventive measures for ASF prevention? (Give a score from not effective/applicable to extremely effective/applicable, 1-5)**

- Training of staff and visitors in biosecurity


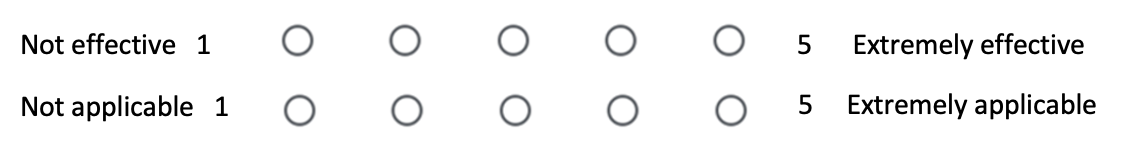


- Restricting movements of people and vehicles into the farm


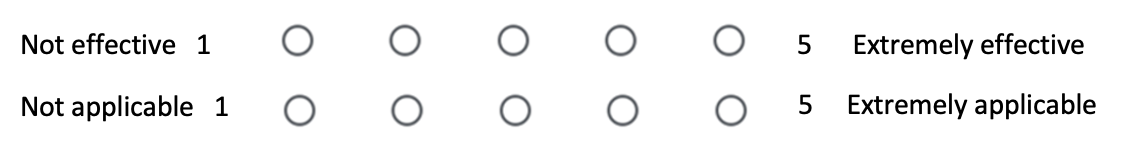


- Fencing


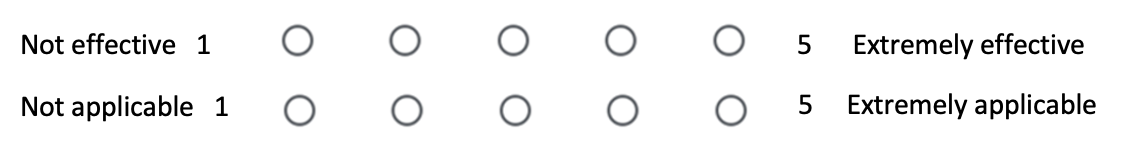


- Double fencing


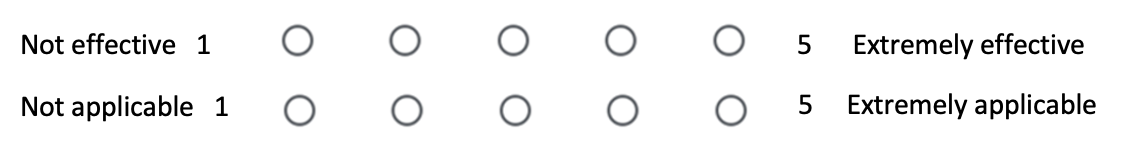


- Confining pig indoor the yard


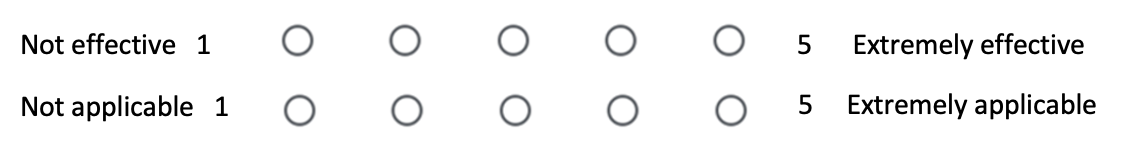


- Ban on going to the forest


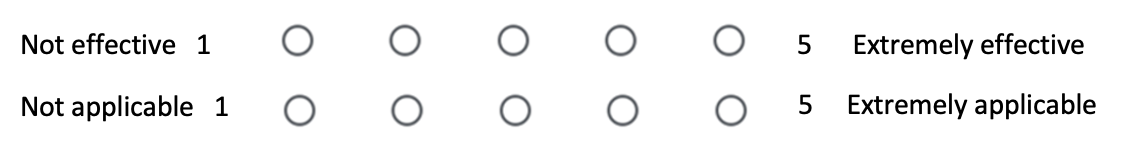


- Washing, changing clothes and footwear


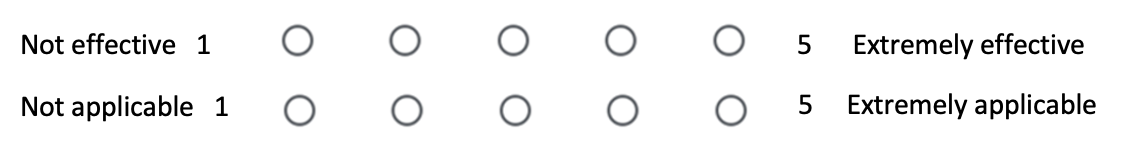


- Rodents control


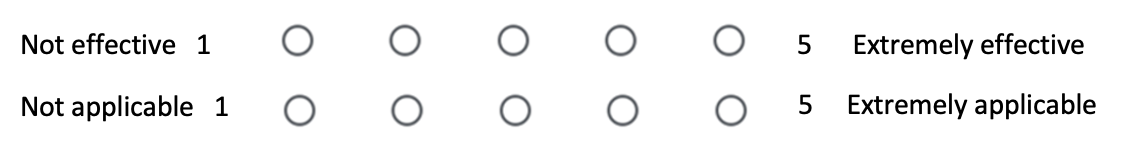


- Forbidding swill feeding (food scraps)


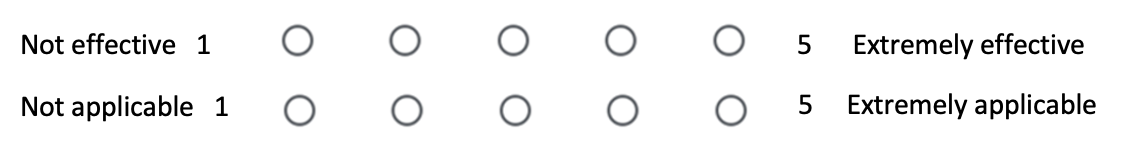


- Outdoor feeding


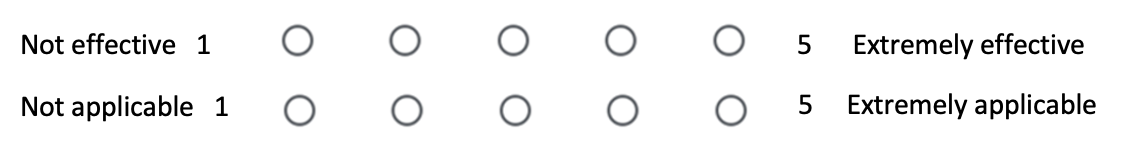


- Cleaning and disinfection of the farm


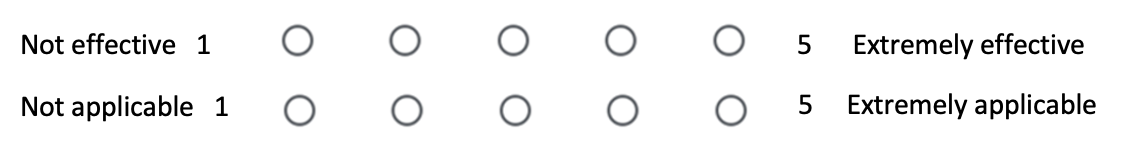


- Farm quarantine


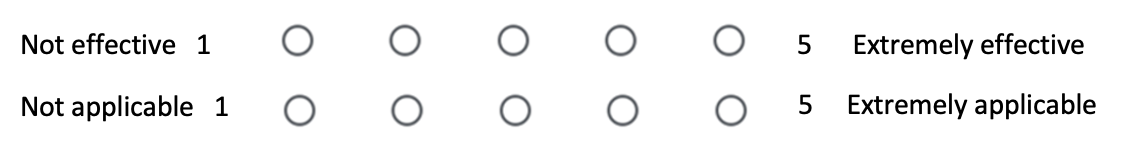


- Destroying the feed and bedding materials on the farm


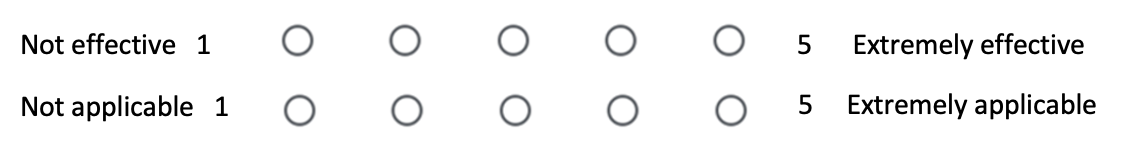


- Culling of all animals on the farm


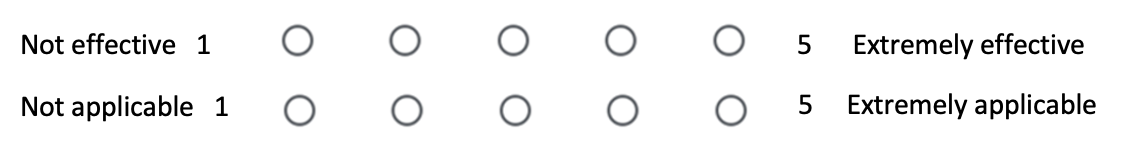


- Using only commercial feed


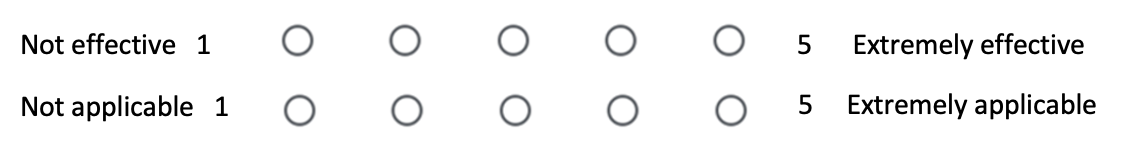


- Other (please specify) ________________________________________________________


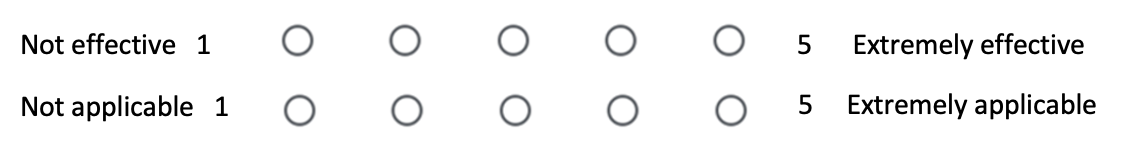


# Farmers’questionnaire template (Bulgarian version)

**ВЛИЯНИЕ НА АЧС ВЪРХУ СТОПАНСТВА
ЗА ОТГЛЕЖДАНЕ НА ИЗТОЧНОБАЛКАНСКА СВИНЯ И ЛИЧНИ СТОПАНСТА „ЗАДЕН ДВОР“
В БЪЛГАРИЯ**

**ИНДИВИДУАЛНИ СЪБЕСЕДВАНИЯ**

Този въпросник цели да събере информация на локално ниво за действащите регулации и политики на ЕС във връзка с АЧС върху стопаните отглеждащи редки породи и собствениците на малки стопанства. Казвам се Елена Лазаро (Elena Lazaro) и работя като ветеринарен лекар към публичен изследователски институт в Италия  (Istituto Zooprofilattico Sperimentale delle Venezie). Имам възможността да получа тази информация благодарение на проект, финансиран от Италианската Сдружение по свиневъдство и патология в партньорство с Институтът по биоразнообразие и екосистемни изследвания при БАН.

Събраните данни ще бъдат използвани в съответствие с Регламент (ЕС) № 2018/1725

Съгласявайки се с декларацията за поверителност на Регламента на ЕС и отговаряйки на този въпросник, вие се съгласявате вашите отговори да се използват анонимно в техническия доклад и свързаната с него публикация за партньорска проверка, която ще бъде изготвена.

- Да, съгласявам се
- Не, не се съгласявам

**ДЕМОГРАФСКИ ДАННИ И ДАННИ ЗА СТОПАНСТВОТО**

***Данни на участниците и общи характеристики на стопанството***

Email ____________________________

Възраст _______________

Пол _______________

Населено място _______________

1. **Настоящ брой на свинете в стопанството ви**

- < 3
- 3-10
- 10-200
- 201-1000
- >1000
- Няма

1. **Какъв е/беше типа на стопанството?**

- Стопанство за Източнобалканска свиня
  - Източнобалкански свине, вписани в родословните книги на породата
  - Кръстоски на ИБС
  - И двете. Посочете съотношение: _________________________________
- Лично стопанство (заден двор) (=*до три прасета за угояване, различни от свине майки и некастрирани нерези*)
- Фамилна ферма (=*до 10 свине майки и техните приплоди, но не повече от 200 прасета общо*)
- Индустриална ферма

**2a. От колко души се състои Вашето домакинство?**

| **Категория** | **Брой** |
| --- | --- |
| Деца <5 годишна възраст |  |
| Деца <18 годишна възраст |  |
| Възрастни >45 годишна възраст |  |
| Възрастни >65 годишна възраст |  |

1. **С каква цел отглеждате свине?**

- лична консумация
- странични доходи, когато е необходимо
- търговска цел (продажба), основен източник на доходи

1. **Какъв тип ферма имате/сте имали?**

- Свободна паша без огради
- Свободна паша в заграждения
  - Как направихте загражденията? ______________________________
  - Вид на загражденията?
    - Дървени заграждения/нецементиран под
    - Дървени заграждения/цементиран под
- Отглеждане на закрито
- Повдигнат дървен под
- Стабилна сграда
- Друго ________________________________

1. **Как храните/хранехте свинете?**

- Отпадъци (хранителни)
  - Преминава ли термична обработка? Да/Понякога/Винаги
- Фураж, трева, зърно и др.
- Горски растения. Какви?___________________________
- Готови специализирани фуражи
- Друго ________________________________

1. **Друг добитък. Имате ли други животни в същата ферма? Ако да, моля, посочете какви.**

- Да __________________________________________________
- Не

1. **Членове на Вашето домакинство упражняват ли дейности, свързани с гората (събиране на гъби, билки, трюфели…)? Ако да, посочете какви и колко често.**
   - Да __________________________________________________
   - Не

**A – ВЛИЯНИЕ НА АЧС ЧЪРХУ СТОПАНСТВОТО ВИ (само за фермери, засегнати от АЧС)**

1. **Имало ли е случай на АЧС във Вашето стопанство? Ако да, посочете месец и година.**

- Да ________________________________________________________________________
- Не

**1.1 Ако да, получили ли сте обезщетения/субсидии за претърпените загуби?**

- Да _______________________________________________________________
- Не

**1.1a Ако да, достатъчна ли беше сумата. Посочете сумата, която сте получили. Каква част от стойността на свинете беше компенсирана?**

_________________________________________________________________

**1.1b Ако не, каква беше причината да не получихте обезщетение/субсидия?**

_________________________________________________________________

1. **Трябваше ли да умъртвите животните си заради това, че сте били близо до друго огнище на АЧС? Ако да, посочете месец и година.**

- Да ____________________________________________________________________
- Не

1. **Приемали ли сте конкретни превантивни мерки във вашата ферма, свързани с АЧС? Ако да, моля, посочете кои (напр. огради), причината и кога.**

- Да ____________________________________________________________________
- Не

**3.1 Получихте ли субсидии за изпълнението на превантивните мерки?**

- Да _______________________________________________________________
- Не

**3.1a Ако да, достатъчна ли беше сумата. Посочете сумата, която сте получили.**

_________________________________________________________________

**3.1b Ако не, каква беше причината да не получихте обезщетение/субсидия?**

_________________________________________________________________

1. **Преминавали ли сте обучения за превенция срещу АЧС във Вашето стопанство?**

- Да ____________________________________________________________________
- Не

1. **Кой беше вашият основен източник на информация за АЧС и да знаете как да действате? (Няколко възможности за избор)**

- Обучителни кампания на ветеринарите
- Брошури и листовки за повишаване на информираността за АЧС
- Местни издания
- Радио
- Телевизионни новини
- Социални мрежи
- Друго (моля уточнете) _____________________________________________________

1. **Кои са наложените ограничения, които според Вас оказаха най-голямо влияние върху Вашата ферма? Моля, мотивирайте отговора си.**

_______________________________________________________________________________________________________________________________________________________________________________________________________________________________________________

1. **Какви бяха негативните последици от АЧС във Вашата ферма? (Няколко възможности за избор)**

- Икономически загуби
- Загуба на работни места
- Несправедливо компенсиране от страна на държавата
- Ограничения при търговията
- Друго (моля уточнете) ______________________________________________________

1. **АЧС оказва ли влияние върху броя на животните във Вашата ферма?**

- Да
  - Може ли да го измерите? _______________________________________________

|  | **Големи** | | **Прасенца** | |
| --- | --- | --- | --- | --- |
|  | **Мъжки** | **Женски** | **Мъжки** | **Женски** |
| Колко прасета имахте преди АЧС? |  |  |  |  |
| Колко прасета имате сега? |  |  |  |  |

- Не

1. **Променили ли сте вида на отглежданите животни или породата свине поради АЧС? Ако да, моля, посочете какви животни/породи сте въвели.**

- Да ____________________________________________________________________
- Не

1. **Променили ли сте навиците си, свързани с гората (напр. лов, бране на гъби...) поради последствия от АЧС? Ако да, уточнете по какъв начин.**

- Да ____________________________________________________________________
- Не

**B – ОЦЕНКА НА ВЪЗПРИЯТИЕТО ЗА АЧС**

1. **Кои са клиничните признаци, които ви карат да подозирате АЧС?**

____________________________________________________________________________________________________________________________________________________________________

1. **Кои са признаците при смърт, които ви карат да подозирате АЧС?**

____________________________________________________________________________________________________________________________________________________________________

1. **В какви случаи бихте повикали ветеринарен лекар за вашите прасета?**

- Едно животно не се храни добре/изглежда болнаво
- Няколко животни не се хранят добре/изглеждат болнави
- Повишена смъртност
- Аборти при свинете-майки или не се заплождат
- Превантивно лечение като ваксинация и/или обезпаразитяване
- Друго________________________________________________________________________

1. **Ако наблюдавате промяна в телесната температура, зачервяване на кожата и смъртност във вашето стадо свине, какво бихте направили?**

- Изчаквам няколко дни, за да видя дали прасетата се подобряват
- Лекувам прасетата с антибиотици
- Продавам прасетата възможно най-скоро
- Обаждам се на ветеринарите
- Друго__________________________________________________________________________

1. **Ако подозирате огнище на АЧС във вашата ферма, какво бихте направили?**

- Изчаквам няколко дни, преди да го докладвам, за да избегна фалшив сигнал
- Изчаквам няколко дни, преди да го докладвам, за да имам време да продам здравите прасета
- Обаждам се възможно най-скоро, дори и да е фалшив случай
- Друго__________________________________________________________________________

1. **Кой начин на предаване на АЧС считате за основен за навлизането ѝ в стопанство?**

____________________________________________________________________________________________________________________________________________________________________

1. **Кой начин на предаване на АЧС считате за основен за разпространението ѝ във вашата страна?**

____________________________________________________________________________________________________________________________________________________________________

1. **Смятате ли, че социалните фактори (напр. движение на хора/животни, споделяне на храна с месни продукти, миграция, пътуване на мигранти) биха могли да играят роля в разпространението на АЧС? Моля, обяснете мнението си.**

____________________________________________________________________________________________________________________________________________________________________

1. **Доколко смятате за ефективни и приложими тези превантивни мерки за предотвратяване на АЧС? (Дайте оценка от неефективни до изключително ефективни, 1-5)**

- Обучение на персонал и посетители по биосигурност


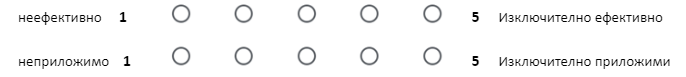


- Ограничаване на движението на хора и превозни средства в стопанството


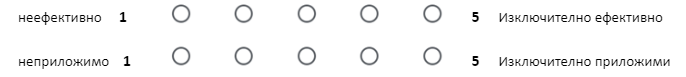


- Поставяне на огради/заграждения

   
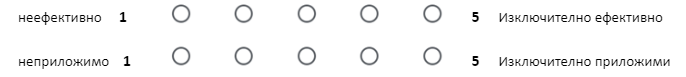


- Двойни огради


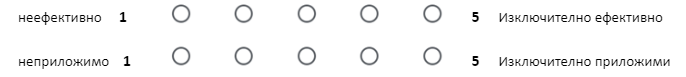


- Затваряне/отглеждане на свинете на закрито в двора


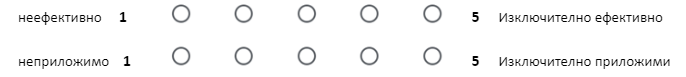


- Забрана за ходене в гората


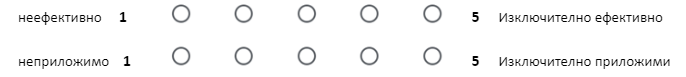


- Измиване и смяна на облекло и обувки


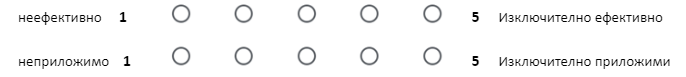


- Контрол на вредителите (гризачи)


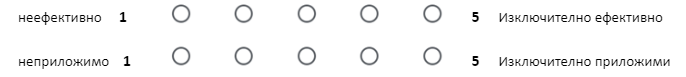


- Забрана за изхранване (на животните) с хранителни отпадъци


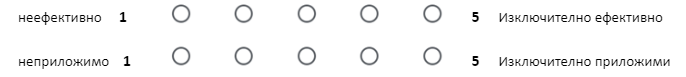


- Хранене на открито


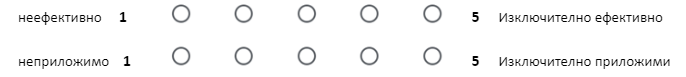


- Почистване и дезинфекция на фермата


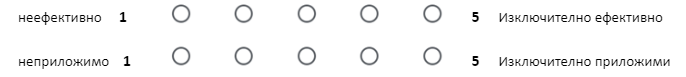


- Карантиниране на фермата


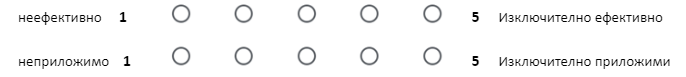


- Унищожаване на фуража и постеля във фермата


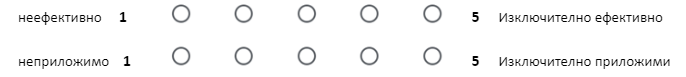


- Умъртвяване на животните в стопанството


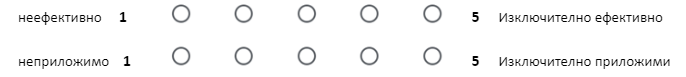


- Използване на готови специализирани фуражи


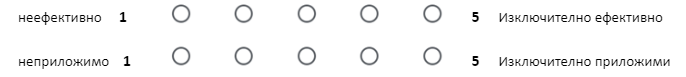


- Друго (моля уточнете) ______________________________________________________


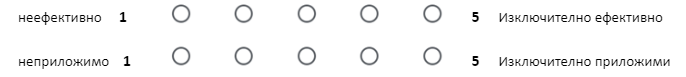


# Non-farmers’ questionnaire template (English version)

**IMPACT OF ASF IN BULGARIA**

**INDIVIDUAL INTERVIEWS**

This questionnaire aims to collect local information on the effect of the current EU Regulations and policies related to ASF on traditional and smallholder farmers. I’m Elena Lazzaro, and I work as a veterinarian in a Public Institute of Research in Italy (Istituto Zooprofilattico Sperimentale delle Venezie). I will collect this data thanks to a project financed by the Italian Society of Swine Farming and pathology, in collaboration with the Institute of Biodiversity and Ecosystem Research.

Data collected will be used according to the Regulation (EU) N° 2018/1725

By agreeing with The EU Regulation privacy statement and answering this questionnaire, you agree that your answers are used anonymously in the technical report and related peer-review publication that will be produced.

- Yes, I Agree
- No, I Don't Agree

**Demographic Data**

Email ___________________

1. **For which organization do you work?** __________________________________________

**A – ASSESSMENT OF ASF PERCEPTION**

1. **Which way of transmission do you consider the most responsible for ASF introduction in a farm?**

________________________________________________________________________________________________________________________________________________________________________________________________________________________________________________________________________________________________________________________________________

1. **Which way of transmission do you consider the most responsible for ASF spread?**

________________________________________________________________________________________________________________________________________________________________________________________________________________________________________________________________________________________________________________________________________

1. **Do you think that social factors (e.g. movement of people/animals, familiar sharing of food meat products, migration, travel migrants) could have played a role in ASF spread? Please explain your opinion.**

________________________________________________________________________________________________________________________________________________________________________________________________________________________________________________________________________________________________________________________________________

1. **How much do you consider effective these preventive measures for ASF prevention? (Give a score from not effective to extremely effective, 1-5)**

- Training of staff and visitors in biosecurity


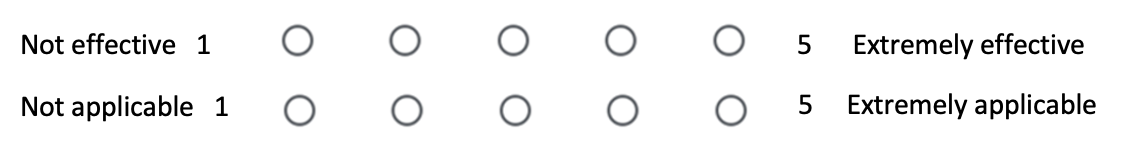


- Restricting movements of people and vehicles into the farm


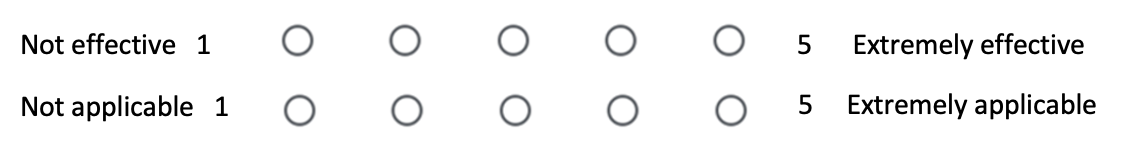


- Fencing


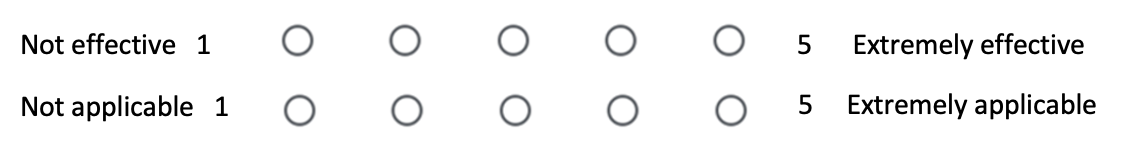


- Ban on going to the forest


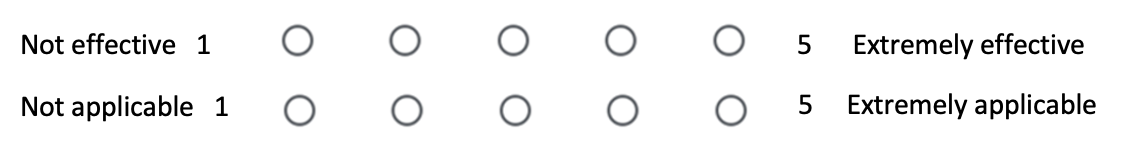


- Washing, changing clothes and footwear


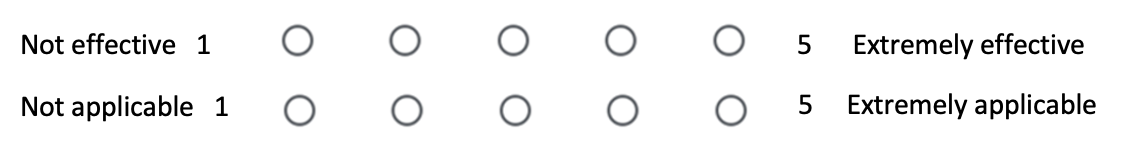


- Rodents control


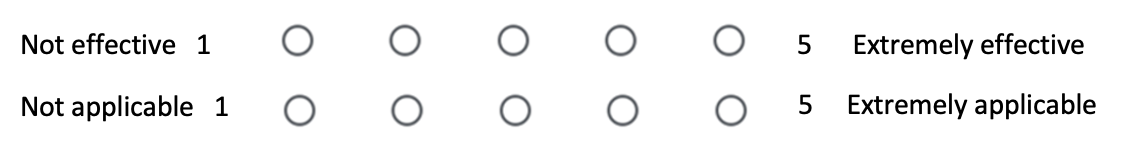


- Forbidding swill feeding


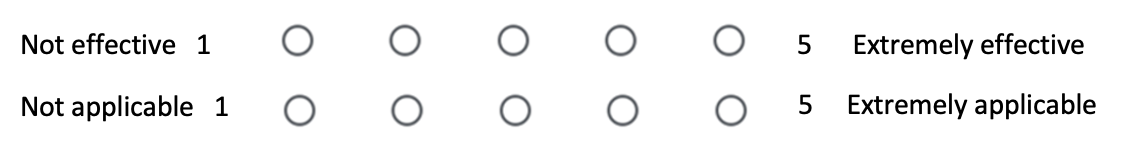


- Outdoor feeding


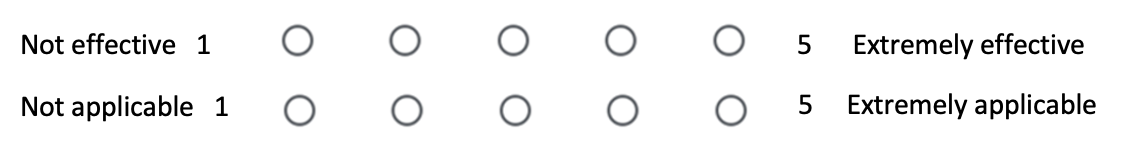


- Other (please specify) ________________________________________________________


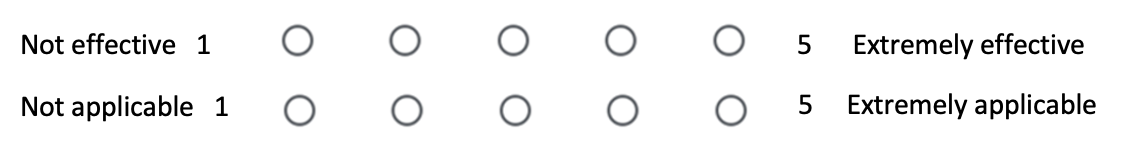


1. **Was there a restriction plan for wild boars? If yes, how was it implemented? Was it effective?**

___________________________________________________________________________________________________________________________________________________________________________________________________________________________________________________________________________

# Non-farmers’ questionnaire template (Bulgarian version)

**ВЛИЯНИЕ НА АЧС В БЪЛГАРИЯ**

**Индивидуални събеседвания**

Този въпросник цели да събере и обобщи информация от първоизточника за ефектите от действащите регулации и политики на ЕС във връзка с АЧС върху стопаните отглеждащи редки породи и собствениците на малки стопанства. Казвам се Елена Лазаро (Elena Lazzaro) и работя като ветеринарен лекар към публичен изследователски институт в Италия (Istituto Zooprofilattico Sperimentale delle Venezie). Имам възможността да получа тази информация благодарение на проект, финансиран от Италианската Сдружение по свиневъдство и патология в партньорство с Институтът по биоразнообразие и екосистемни изследвания при БАН.

Събраните данни ще бъдат използвани в съответствие с Регламент (ЕС) № 2018/1725

Съгласявайки се с декларацията за поверителност на Регламента на ЕС и отговаряйки на този въпросник, вие се съгласявате вашите отговори да се използват анонимно в техническия доклад и свързаната с него публикация за партньорска проверка, която ще бъде изготвена.

- Да, съгласявам се
- Не, не се съгласявам
- **Демографски данни**

Email ___________________

1. **За коя организация работите?** __________________________________________

**A – ОЦЕНКА НА ВЪЗПРИЯТИЕТО ЗА АЧС**

1. **Кой начин на предаване на АЧС считате за основен за навлизането ѝ в стопанство?**

________________________________________________________________________________________________________________________________________________________________________________________________________________________________________________________________________________________________________________________________________

1. **Кой начин на предаване на АЧС считате за основен за разпространението ѝ?**

________________________________________________________________________________________________________________________________________________________________________________________________________________________________________________________________________________________________________________________________________

1. **Смятате ли, че социалните фактори (напр. движение на хора/животни, споделяне на храна с месни продукти, миграция, пътуване на мигранти) биха могли да играят роля в разпространението на АЧС? Моля, обяснете мнението си.**

________________________________________________________________________________________________________________________________________________________________________________________________________________________________________________________________________________________________________________________________________

1. **Доколко смятате за ефективни тези превантивни мерки за предотвратяване на АЧС? (Дайте оценка от неефективни до изключително ефективни, 1-5)**

- Обучение на персонал и посетители по биосигурност


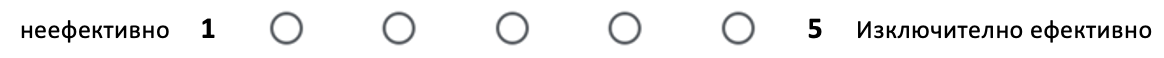


- Ограничаване на движението на хора и превозни средства в стопанството


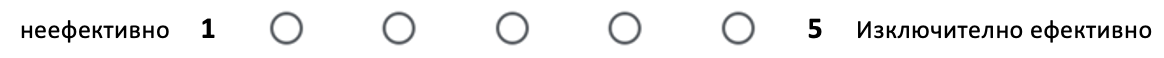


- Поставяне на огради


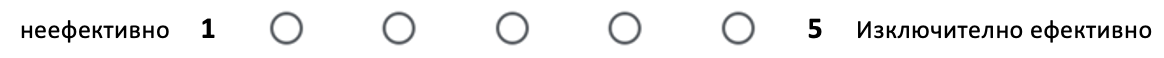


- Забрана за ходене в гората


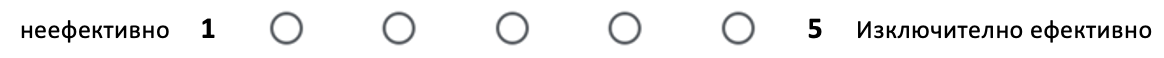


- Измиване и смяна на облекло и обувки


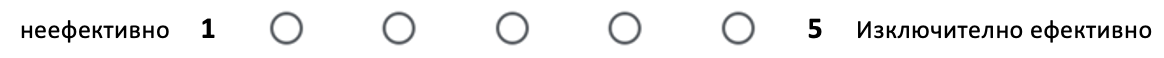


- Контрол на вредителите (гризачи)


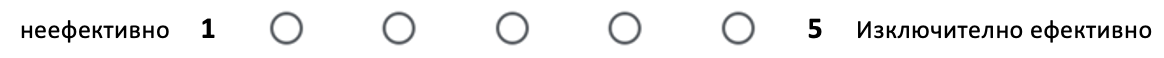


- Забрана за изхранване (на животните) с хранителни отпадъци


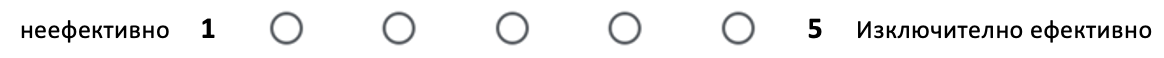


- Хранене на открито


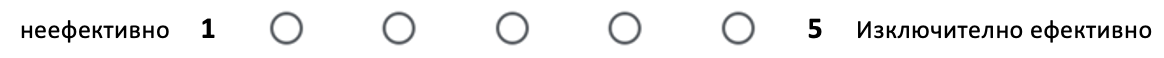


- Друго (моля опишете) ________________________________________________________


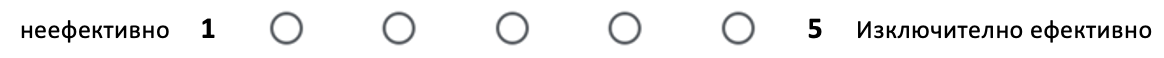


1. **Имаше ли план за ограничаване на дивите свине? Ако да, как е приложен? Ефективно ли беше?**

___________________________________________________________________________________________________________________________________________________________________________________________________________________________________________________________________________
